# Enhanced Performance by Interpretable Low-Frequency Electroencephalogram Oscillations in the Machine Learning-Based Diagnosis of Post-traumatic Stress Disorder

**DOI:** 10.3389/fninf.2022.811756

**Published:** 2022-04-26

**Authors:** Miseon Shim, Chang-Hwan Im, Seung-Hwan Lee, Han-Jeong Hwang

**Affiliations:** ^1^Department of Electronics and Information, Korea University, Sejong, South Korea; ^2^Industry Development Institute, Korea University, Sejong, South Korea; ^3^Department of Biomedical Engineering, Hanyang University, Seoul, South Korea; ^4^Department of Psychiatry, Ilsan Paik Hospital, Inje University, Goyang, South Korea; ^5^Clinical Emotion and Cognition Research Laboratory, Goyang, South Korea; ^6^Interdisciplinary Graduate Program for Artificial Intelligence Smart Convergence Technology, Korea University, Sejong, South Korea

**Keywords:** machine-learning technique, classification, computer-aided diagnosis, resting-state electroencephalogram (EEG), slow-frequency EEG oscillation, post-traumatic stress disorder (PTSD)

## Abstract

Electroencephalography (EEG)-based diagnosis of psychiatric diseases using machine-learning approaches has made possible the objective diagnosis of various psychiatric diseases. The objective of this study was to improve the performance of a resting-state EEG-based computer-aided diagnosis (CAD) system to diagnose post-traumatic stress disorder (PTSD), by optimizing the frequency bands used to extract EEG features. We used eyes-closed resting-state EEG data recorded from 77 PTSD patients and 58 healthy controls (HC). Source-level power spectrum densities (PSDs) of the resting-state EEG data were extracted from 6 frequency bands (delta, theta, alpha, low-beta, high-beta, and gamma), and the PSD features of each frequency band and their combinations were independently used to discriminate PTSD and HC. The classification performance was evaluated using support vector machine with leave-one-out cross validation. The PSD features extracted from slower-frequency bands (delta and theta) showed significantly higher classification performance than those of relatively higher-frequency bands. The best classification performance was achieved when using delta PSD features (86.61%), which was significantly higher than that reported in a recent study by about 13%. The PSD features selected to obtain better classification performances could be explained from a neurophysiological point of view, demonstrating the promising potential to develop a clinically reliable EEG-based CAD system for PTSD diagnosis.

## Introduction

Post-traumatic stress disorder (PTSD) is a psychiatric disorder caused by experiencing or witnessing traumatic events (Yehuda, 2002), and PTSD patients are diagnosed through interview with clinical experts based on the Diagnostic and Statistical Manual of Mental Disorders, 5th Edition (DSM-5, note that all abbreviations were summarized in Supplementary Table 1) (Friedman et al., 2011). Post-traumatic stress disorder patients generally show a high rate of comorbidity with other mental illnesses, which leads to confusion of diagnosis (Ginzburg et al., 2010; Elhai et al., 2011; Gros et al., 2012). According to a previous study, 73.3% of veteran PTSD patients had comorbid anxiety disorders (e.g., general, panic, and social anxiety disorder) (Magruder et al., 2005). Another study reported that 68% of veteran PTSD patients met the criteria for major depressive disorder (MDD) (Grubaugh et al., 2010). These results imply that when interviewing with clinical experts, if PTSD patients hide their traumatic histories or symptoms, they could be misdiagnosed as other mental diseases with high probability. Thus, it is necessary to introduce a PTSD diagnosis tool to compliment the diagnostic failure rate of traditional diagnosis (Sumpter and McMillan, 2005).

In recent years, the machine-learning-based computer-aided diagnosis (CAD) system has received increased attention, due to its ability to predict the state of neuropsychiatric diseases using objective neurophysiological biomarkers (McBride et al., 2011; Mitra et al., 2016; Vergara et al., 2017, 2018). Early CAD systems developed for those suffering from traumatic events have used neuroimaging-based features for its diagnosis, but they have focused on patients with traumatic brain injury (TBI), rather than PTSD (McBride et al., 2011; Mitra et al., 2016; Vergara et al., 2017, 2018). For example, Mitra et al., achieved 68% classification accuracy when differentiating TBI from healthy controls (HC) using diffusion tensor imaging (DTI) features (Mitra et al., 2016), meanwhile, Vergara et al., reported a classification accuracy of 84% when differentiating TBI patients from HC using DTI and functional magnetic resonance imaging (fMRI)-based features (Vergara et al., 2017). However, although both TBI and PTSD are developed by traumatic events, TBI and PTSD should be studied independently because the cause and characteristics of TBI and PTSD are totally different, in that TBI patients are troubled with physical brain damage, while PTSD patients suffer from mental problems (Bryant, 2011).

A few studies have attempted to differentiate PTSD patients from HC using neuroimaging-based features. Rangaprakash et al. (2017) achieved 81% classification accuracy when classifying PTSD patients and HC using effective connectivity network features extracted from resting-state fMRI data. In addition, Zhang et al., introduced a classification model for PTSD diagnosis using magnetoencephalographic (MEG) connectomes, and reported the performance of an area-under-the-curve (AUC) value of 0.9 (Zhang et al., 2020). However, while fMRI and MEG have shown the potential to differentiate PTSD patients from HC, due to their high cost and low portability, they are not usable in practice for both clinicians and patients (Wienbruch et al., 2006). To overcome the mentioned limitations, electroencephalogram (EEG) could be an adequate alternative neuroimaging tool for the diagnosis of PTSD patients (Chen, 2001).

Electroencephalogram (EEG) is more portable than other neuroimaging tools, such as fMRI and MEG (Chen, 2001), and is also suitable to investigate dynamic neuronal changes due to its high-temporal resolution (Saletu et al., 1991; Burle et al., 2015). In particular, since abnormal neuronal changes in psychiatric patients reflect their pathophysiology (Newson and Thiagarajan, 2019), they could be used for the diagnosis of various psychiatric diseases (Orru et al., 2012). Therefore, many researchers have introduced EEG-based CAD systems for the diagnosis of various psychiatric disorders, and achieved promising classification accuracies when differentiating psychiatric disorders from HC (Orru et al., 2012). Recently, we introduced 2 EEG-based CAD systems to assist the accurate diagnosis of PTSD patients (Shim et al., 2019; Kim Y. W. et al., 2020).

In our previous study, we attempted to classify PTSD patients and HC using P300 event-related potential (ERP) based on machine-learning technique (Shim et al., 2019), and obtained a classification accuracy of 80% in differentiating 2 groups. Although we achieved acceptable classification accuracy, the previous study was limited in terms of its usability; PTSD patients were required to perform an auditory attention task to evoke P300 activation even though they generally have difficulty in concentrating on an attention-related task. To overcome this limitation, resting-state EEG could be an appropriate alternative because no effort is required to record resting-state EEG from PTSD patients; also, resting-state EEG reflects the pathophysiological traits of PTSD patients (McFarlane et al., 2005; Veltmeyer et al., 2006). Recently, we investigated the possibility of using resting-state EEG to distinguish PTSD patients from HC, where we employed 2 types of source-level features: (i) power spectrum densities (PSDs) and (ii) network indices based on graph theory (Kim Y. W. et al., 2020). We confirmed that PSD features showed significantly higher classification accuracy than network features, and obtained a maximum classification accuracy of 73.09%.

The objective of this study was to enhance the performance of classifying PTSD patients from HC by optimizing the frequency band used for extracting PSD features from resting-state EEG. Our previous study (Kim Y. W. et al., 2020) did not consider a delta-frequency band of 1–4 Hz even though slow EEG waves were closely related to the typical endophenotypes of PTSD patients (McFarlane et al., 2005; Veltmeyer et al., 2006; Newson and Thiagarajan, 2019), and an optimization of frequency bands was also not performed using traditional 6 frequency bands (delta, theta, alpha, low-beta, high-beta and gamma) in terms of classification performance. Therefore, in this study, we aimed to optimize PSD frequency bands by introducing the delta band and investigating all possible combinations of the 6 frequency bands in order to increase the classification performance between PTSD patients and HC. Furthermore, we investigated the neurophysiological meanings of most discriminable features selected to obtain the best classification performance, thereby contributing to the development of a reliable CAD system to assist the diagnosis of PTSD patients. The main highlights of our contributions are briefly listed below:

i) Optimization of resting-state EEG-based features for assisting diagnosis of PTSD patients.
ii) Significant improvement of classification performance as compared to a recent study by about 13%.
iii) Interpretation of useful features selected to attain the best classification performance from a neurophysiological point of view, thereby providing the basis to develop a reliable CAD system for PTSD diagnosis.

## Materials and Methods

### Participants

Seventy-seven PTSD patients aged between 20 and 60 years and 58 HC aged between 23 and 60 years were recruited for this study from the Psychiatry Department of Inje University Ilsan Paik Hospital (see Table 1 for detailed demographic data). The patients’ diagnosis by a board-certified psychiatrist was based on the Diagnostic and Statistical Manual of Mental Disorders, 4th edition (DSM-IV) Axis I Psychiatric Disorders. Patients were excluded if they accorded with the following criteria: (1) abnormality of the central nervous system, (2) medical histories of alcohol or drug abuse, (3) intellectual disability, (4) history of head injuries with loss of consciousness and experience with electrical therapy (e.g., electroconvulsive therapy, ECT), and (5) psychotic symptoms lasting for at least 24 h. HC was recruited from the local community through local newspapers and posters. Individuals without any psychiatric medical history were recruited for HC. If the HC was taking or had taken any kinds of psychotropic medication, they were excluded from the study. The study protocol was approved by the Institutional Review Board of Inje University Ilsan Paik Hospital (2015-09-018), and all participants provided written informed consent.

**TABLE 1:** Demographic data of post-traumatic stress disorder (PTSD) patients and healthy controls (HC). The *p-*values represent significant differences between the two groups.

	PTSD	HC	*p*-value
Cases (*N*)	77	58	
Gender (male/female)	28/49	30/28	0.082
Age (years) Range	40.92 ± 11.93 20 – 60	39.98 ± 11.63 23 – 60	0.646
Education	13.51 ± 2.80	14.45 ± 3.37	0.120
IES-R	51.34 ± 21.71		
BDI	26.99 ± 13.13		
BAI	29.48 ± 15.44		

*IES-R, Impact of Event Scale-Revised; BAI, Beck Anxiety Inventory; BDI, Beck Depression Inventory. The p-values are obtained using an independent t-test for age and education, and chi-squared test for gender.*

Three psychiatric symptoms of PTSD patients were evaluated by clinical experts. Impact of Event Scale-Revised (IES-R) (Weiss, 2007) was used to determine whether the patients had PTSD or not by evaluating the response severity of traumatic events. Beck Anxiety Inventory (BAI) (Beck and Steer, 1990) and Beck Depression Inventory (BDI) (Beck et al., 1996) were used to check anxiety symptom and depression symptom, respectively (Table 1). Three types of psychotropic medications were prescribed to the patients based on patients’ clinical symptoms examined by clinical experts: antidepressants for depressive symptoms [selective serotonin reuptake inhibitors (*n* = 67), venlafaxine (*n* = 10)], antipsychotics for psychotic symptoms [aripiprazole (*n* = 5), quetiapine (*n* = 17)], and sedative-hypnotics for anxiety symptoms [lorazepam (*n* = 37), clonazepam (*n* = 27), diazepam (*n* = 15), and alprazolam (*n* = 35)]. All patients received combined psychotropic medications as follows: (1) antidepressant, antipsychotics, and sedative-hypnotics (*n* = 32); (2) antidepressant and antipsychotics (*n* = 12); (3) antidepressant and sedative-hypnotics (*n* = 33).

### Electroencephalogram Recording and Preprocessing

Resting-state EEG data used in this study were the same as presented in our previous study (Shim et al., 2017), where we only investigated disrupted brain networks and the relationships between network indices and symptoms of PTSD patients. Resting-state EEG data were recorded with a band-pass filter of 1–100 Hz for 5 min with eyes closed (sampling rate: 1,000 Hz), for which 64 Ag/AgCl scalp electrodes were evenly mounted on the scalp according to the extended international 10-20 system [NeuroScan SynAmps2 (Compumedics United States, El Paso, TX, United States); references: M1 and M2]. To reduce both external and internal artifacts of EEG data, a series of pre-processing approaches were applied to raw EEG data. Firstly, external artifacts, such as electrocardiography (ECG) and eye-related artifacts (e.g., eyes blinks and horizontal movements), were removed using established mathematical procedures based on regression approach (Semlitsch et al., 1986), and other gross artifacts (e.g., if electrodes showed amplitudes higher than 200 μV) were rejected by visual inspection. To correct the baseline, the DC offset of EEG channels was removed by subtracting the average values of EEG data from each time point for each channel. The baseline corrected EEG data were then band-pass filtered between 1 and 55 Hz using a third order Butterworth IIR filter to remove high-frequency external artifacts, and they were epoched with a length of 4.096 s. Epochs were rejected if they contained significant physiological artifacts (± 100 μV) at any electrode (Kim et al., 2019), and 10 artifact-free epochs extracted for each subject were used for further analysis, as in previous studies (Gudmundsson et al., 2007; Shim et al., 2017). Since a previous study reported that a total of 40 s epoch is sufficient to obtain reliable results of quantifying resting-state EEG data, we used 10 epochs (40.96 s) to extract classification features from resting-state EEG (Gudmundsson et al., 2007).

### Source-Level Power Spectrum Densities Feature Extraction

Because related studies reported the superiority of source-level features as compared to sensor-level features (Shim et al., 2016, 2019; Kim J. et al., 2020) as well as of PSD features as compared to network features in terms of classification performance (Kim Y. W. et al., 2020), we used source-level PSD features for the classification of PTSD patients from HC. To estimate the source-level time series, a lead-field matrix was computed using a three-layer (inner skull, outer skull, and scalp) boundary element method (BEM) model, which was constructed from the standard head model (Colin 27) using the OpenMEEG toolbox (Gramfort et al., 2010). An inverse operator was created using a weighted minimum-norm estimation (wMNE) algorithm implemented in Brainstorm toolbox (Tadel et al., 2011). A time-series of source activities at 15,000 cortical vertices was estimated for every time point using the EEG data created by concatenating 10 artifact-free EEG epochs to improve computational efficacy (Kang et al., 2018). After computing current source densities, representative source signals at 68 regions of interests (ROIs) based on the Desikan–Killiany atlas were estimated using the 1*^st^* component of principal component analysis (PCA) (Dimitriadis et al., 2018). We excluded 18 ROIs that showed statistical difference between 2 groups (PTSD vs. HC) in terms of the variance explained by the 1*^st^* PC because in this case the 1*^st^* PCs of 2 groups did not similarly explain the variance of original data (independent *t*-test; Bonferroni corrected *p* < 0.05). Therefore, the source signals at the remaining 50 ROIs were used for further data analysis, and the source signals of each ROI was epoched into 4.069 s. Supplementary Table 2 provides the name of all 68 ROIs, their corresponding variances explained by the 1*^st^* PCs, and statistical test results, respectively. Time-varying source-level PSDs were then estimated by a complex Morlet-Wavelet method using “ft_freqanalysis” Matlab function implemented in Fieldtrip toolbox (Oostenveld et al., 2011). A crucial input parameter “cgf.width” in “ft_freqanalysis” was set as 3 to appropriately determine a wavelet width (= cfg.width/frequency/pi) according to the recommendation of the Fieldtrip guideline, thereby guaranteeing the accurate estimation of PSDs for all frequencies with the EEG epoch of 4.096 s. The Morlet-wavelet transform with a sinusoidal wave modified by a Gaussian shape was applied to the source-level time series of each ROI. Source-level PSDs of each ROI were independently quantified by averaging time-varying PSDs in 6 frequency bands, i.e., delta [1–4 Hz], theta [4–8 Hz], alpha [8–12 Hz], low-beta [12–22 Hz], high-beta [22–30 Hz], and gamma [30–55Hz]. Note that the beta-frequency band was divided into 2 sub-bands: low-beta [12–22 Hz] and high-beta [22–30 Hz] bands (Kim et al., 2018; Shim et al., 2018). 30 PSD feature sets were finally constructed by integrating the PSD features of different frequency bands (Table 2).

**TABLE 2:** Thirty power spectrum density (PSD) feature sets constructed by combining different frequency bands and the number of features for each feature set. Fifty features were extracted for each frequency band.

Frequency bands	The number of features
D, T, A, LB, HB, G	50 ROIs × 1 frequency band = 50
D + T, D + A, D + LB, D + HB, D + G, T + A, T + LB, T + HB, T + G, A + LB, A + HB, A + G, LB + HB, LB + G, HB + G	50 ROIs × 2 frequency bands = 100
D + T + A, T + A + LB, A + LB + HB, LB + HB + G	50 ROIs × 3 frequency bands = 150
D + T + A + LB, T + A + LB + HB, A + LB + HB + G	50 ROIs × 4 frequency bands = 200
D + T + A + LB + HB	50 ROIs × 5 frequency bands = 250
D + T + A + LB + HB + G	50 ROIs × 6 frequency bands = 300

*D, delta; T, theta; A, alpha; LB, low-beta; HB, high-beta; G, gamma.*

### Classification

**FIGURE 1:**
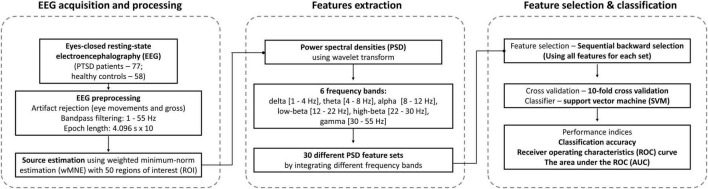
Flowchart of machine-learning-based classification approach.

30 PSD feature sets were independently tested to evaluate the performance of classifying PTSD patients and HC, thereby finding an optimal combination of PSD frequency bands with respect to classification performance. To this end, classification performances were evaluated using the features by sequentially eliminating recursive features from all features for each feature set using sequential backward selection (SBS) method (García-Laencina et al., 2014). The classification accuracy was evaluated using a linear support vector machine (SVM) classifier (Orru et al., 2012; Alimardani et al., 2018) with a 10-fold cross validation method to prevent overestimate classification performance and improve computation efficacy for each of the 30 feature sets (Kim Y. W. et al., 2020). Note that we also tested 2 other machine-learning classifiers, Random Forest and AdaBoost, as well as 3 deep learning classifiers based on convolutional neural network (CNN), shallow ConvNet (Schirrmeister et al., 2017), EEGNet (Lawhern et al., 2018), and a 13 layers-based deep CNN (Acharya et al., 2018), but we only report SVM results due to its superior classification performance as compared to the other classifiers. In this study, the number of subjects (sample size) in each group was imbalanced (PTSD – 77 and HC – 58), which could lead to biased classification performances. To complement the effect of the imbalance sample size on classification, we employed 2 strategies: (1) a cost-sensitive SVM classifier that modifies the weight of margin penalty proportional to sample size and (2) a balanced classification accuracy [sensitivity + specificity)/2]. Moreover, we evaluated the receiver operating characteristics (ROC) curve and the area under the ROC (AUC) as another performance measure (Long et al., 2017). In addition, to investigate what features (ROIs) were most importantly used for classification, SVM coefficients were evaluated for each ROI (Bosilj et al., 2018) when maximum classification accuracies were obtained for 2 best feature sets extracted from delta and theta frequency bands. Since different features were selected within each cross-validation loop, the absolute values of SVM coefficients were averaged for each of the ROIs selected across cross validation, and they were normalized between 0 and 1. Then, we visualized the ROIs with different sizes and colors along with PSD features where the size of ROIs was proportional to SVM coefficients. A higher SVM coefficient (lager circle) means higher importance in terms of classification based on which neurophysiological interpretation is possible (Bosilj et al., 2018). Figure 1 represents the flowchart of this study.

## Results

### Classification Accuracy

**FIGURE 2:**
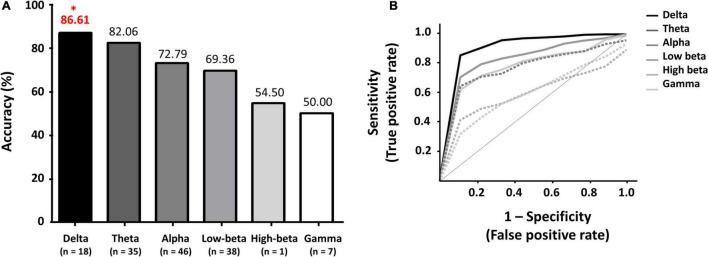
**(A)** Maximum classification accuracies of each frequency band, and **(B)** Receiver operating characteristic (ROC) curves with corresponding areas under the curves (AUC) for each frequency band. *indicates a maximum classification accuracy.

Figures 2A,B show the maximum classification accuracies of each frequency band and the corresponding ROC curves with AUC values, respectively: delta: 86.61% with 0.93 (AUC); theta: 82.06% with 0.86; alpha: 72.79% with 0.80; low-beta: 69.36% with 0.79; high-beta: 54.50% with 0.56; and gamma: 50.00% with 0.56 (see Supplementary Table 3 for the balanced classification accuracies of all feature sets). The delta and theta feature sets only showed acceptable classification performance for a practical binary classification system (> 70%) (Perelmouter and Birbaumer, 2000; Hwang et al., 2014). The combinatory feature set of delta and theta features showed almost same classification performance with the delta feature set (86.39%). Other combinatory feature sets, including the delta feature set, also showed comparable classification performance with the delta feature set, e.g., 86.17% for delta + alpha, delta + low-beta, and delta + high-beta, and 85.96% for delta + theta + alpha + low-beta. Note that no feature sets outperformed the delta feature set in terms of classification performance (Supplementary Table 3).

### Spatial Power Spectrum Density Distribution and Important Features

**FIGURE 3:**
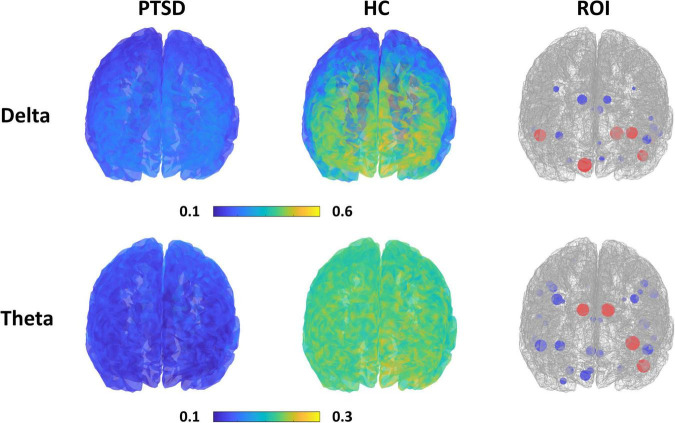
Spatial PSD distributions for each group (first and second column) with respect to the frequency band, and the ROIs selected when achieving the maximum classification accuracy for each frequency band (third column). Red circles represent the important ROIs that have SVM coefficients over the upper bound of 95% confidence interval and blue circles indicate the other selected ROIs. The size of circles is proportional to SVM coefficients.

Figure 3 presents the spatial PSD distributions of delta and theta bands, respectively, and ROIs selected when achieving the maximum classification accuracies. Overall, the PSDs of PTSD patients were considerably reduced, as compared to those of HC (first and second columns of Figure 3). Red circles represent the important ROIs that have SVM coefficients over the upper bound of 95% confidence interval (mean + 2 standard deviation) and blue circles indicates the other selected ROIs. Most of the selected ROIs were overlapped between the 2 frequency bands and they were located in the fronto-temporal area.

## Discussion

In the present study, we investigated the optimal PSD frequency bands to improve the performance of a resting-state EEG-based CAD system to assist the diagnosis of PTSD patients using machine-learning technique. The classification accuracies of the lower frequency PSD feature sets (delta and theta) were significantly higher than those of the relatively higher frequency PSD feature sets (alpha, low-beta, high-beta, and gamma). The best classification performance was obtained when using delta PSDs (86.61% and AUC – 0.93). The features that were selected to attain the best classification accuracy were closely related to the neurophysiological characteristics of PTSD patients, which will be discussed in detail.

### Source-Level Power Spectrum Density Features for the Classification of Post-traumatic Stress Disorder Patients

It has been well documented that the results of sensor-level analysis would be distorted and smeared by volume conduction effects due to different tissue conductivities (van den Broek et al., 1998), and thereby sensor-level analysis could not be used to accurately extract neuronal information (Babiloni et al., 1997). The introduction of source-level analysis could redeem the weaknesses of sensor-level analysis (Michel et al., 2004). In fact, many researchers have utilized source-level features to develop CAD systems to assist the diagnosis of psychiatric disorders. For example, previous EEG and MEG studies showed higher classification accuracies when using source-level features than sensor-level features to differentiate schizophrenia patients from HC (Shim et al., 2016; Kim J. et al., 2020), PTSD patients from HC (Shim et al., 2019; Kim Y. W. et al., 2020), and PTSD patients from major depressive disorder patients (Shim et al., 2019). Therefore, in this study, we only considered source-level features to classify PTSD patients from HC rather than using sensor-level analysis, and achieved reasonable classification performance.

Power spectrum densities (PSDs) have been mainly used as a classification feature type to develop a CAD system to assist in the diagnosis of psychiatric patients (Cassani et al., 2018; Radenković and Lopez, 2019) because distinct and abnormal PSD patterns were shown in psychiatric disorders, as compared to HC (Newson and Thiagarajan, 2019); these abnormal PSD patterns were also related to genetic traits of psychiatric disorders (Venables et al., 2009). PTSD patients also showed altered PSD patterns compared to HC; PTSD patients revealed significantly diminished PSDs in delta and theta bands (McFarlane et al., 2005; Veltmeyer et al., 2006; Newson and Thiagarajan, 2019). Moreover, the diminished PSDs in slow-frequency bands significantly correlated with patients’ symptom scores (Clinician-Administered PTSD scale, CAPS) (Veltmeyer et al., 2006), indicating that the altered PSD patterns would reflect the pathophysiology of PTSD patients, such as re-experiencing and arousal (Veltmeyer et al., 2006). Abnormal PSD patterns of PTSD patients were also clearly observed in this study (Figure 3), and they played an important role in classifying PTSD patients from HC. However, unfortunately, we did not find any significant relationship between the PSD features used to obtain a relatively high performance (i.e., 18 features for delta band and 35 features for theta band) and symptom scores (i.e., IES-R, BAI, and BDI) in this study. This would be because these features were selected from a machine learning perspective for better classification; low frequency PSD features for each group (PTSD and HC) were separately grouped with a discriminable distance (i.e., relatively high PSD values for HC vs. low PSD values for PTSD, as shown in Figure 3), but which does not guarantee that the low frequency PSD features of PTSD patients are necessarily correlated with neuropsychological estimates (e.g., symptom scores). Note that the discriminable low frequency PSD features were selected by comparing those of PTSD patients and HC, but symptom scores were acquired only from PTSD patients. Because of the mentioned reason, the low frequency PSD features of PTSD patients did not seem to be proportional to symptom scores even though the low frequency PSD features can be used for the discrimination of PTSD patients from HC. In order to find a certain feature set that is directly correlated with neuropsychological estimates, regression should be used instead of classification by focusing on only PTSD data, but which would be beyond the scope of this study, focusing on the discrimination of 2 groups. Nevertheless, it is important to investigate neuropsychological traits using objective neurophysiological biomarkers in order to improve the understanding of neural mechanism in psychiatric disorders. Therefore, we will keep going to develop a new feature-based CAD system that can provide both the accurate diagnosis of psychiatric patients in terms of machine learning and the understanding of neurophysiological traits in terms of neuroscience by adopting other EEG-based metrics, such as effective connectivity and complexity measures.

### Crucial Role of Slow Brain Waves

In the present study, the best classification performance was obtained when using delta PSD features. The best classification performance obtained in this study (86.61%) was considerably higher than that of our previous PTSD study by approximately 13% (Kim Y. W. et al., 2020). Unlike the previous study, we used delta PSD features when differentiating PTSD patients and HC, which mainly led to the relatively higher classification accuracy. Moreover, when using theta PSD features, a reasonable classification accuracy of 82.06% was also achieved, which was also higher than that of the previous study (Kim Y. W. et al., 2020). The performance improvement would be caused by the distinct characteristics of slow EEG waves of PTSD patients in the delta and theta frequency bands. Many previous studies reported that slow EEG waves were a typical endophenotype of PTSD patients (Franke et al., 2016; Sheerin et al., 2018; Newson and Thiagarajan, 2019). As mentioned above, PTSD patients showed significantly decreased delta and theta PSDs compared to HC, and the decreased slow-frequency PSDs were closely associated with their altered symptoms, such as arousal and numbing (Franke et al., 2016; Sheerin et al., 2018). That is, due to the distinct pathological traits of slow EEG waves in PTSD patients, the present study could significantly improve the classification performance for the diagnosis of PTSD patients, compared to that of our previous study (Kim Y. W. et al., 2020).

### Spatial Distribution of Power Spectrum Density Features

All PSD features selected when attaining maximum classification accuracies for delta and theta bands were extracted from fronto-temporal areas, such as frontal pole, opercular, anterior cingulate gyrus, superior temporal gyrus, and temporal pole (third column of Figure 3). The mean PSD values of each ROI of the mentioned fronto-temporal areas were significantly smaller in PTSD patients than those in HC. According to previous studies, the frontal regions, including the frontal pole and opercular part of the inferior frontal area, are involved in emotion regulation processing (Grecucci et al., 2013; Bramson et al., 2019) and temporal areas are significantly related to rumination symptom of PTSD patients (Ferdek et al., 2016); PTSD patients showed significantly reduced brain activation in both brain areas in resting-state (Rabe et al., 2006), as shown in this study. Therefore, it is neurophysiologically plausible to obtain better classification performance for PTSD diagnosis when using PSD features extracted from fronto-temporal areas. This also indicates that employing only fronto-temporal areas might be sufficient to implement a reliable CAD system for PTSD diagnosis, thereby facilitating the development of a more clinically practical CAD system.

In this study, we used EEG data measured in resting state during which default mode network (DMN) or salience network is more active than the task period (Choi et al., 2021), and thus DMN might be usefully used for PTSD diagnosis. We investigated the ROIs selected when the maximum classification accuracy was obtained using delta PSD features, and found that 16 of 18 DMN ROIs were selected together with 2 other ROIs, i.e., entorhinal and para hippocampal areas. Both entorhinal and para hippocampal areas are known to be related to clinical symptoms in PTSD patients, such as altered cognitive function and disrupted memory (Kerr et al., 2007). This result means that it is necessary to use the features extracted from the ROIs related to neuropsychological traits of patients together with those related to current brain state (resting state in this study) to obtain a relatively high diagnosis accuracy.

### Machine Learning Approach With Interpretable Electroencephalogram Biomarkers

In recent years, machine learning approaches have received increasing attention in the development of an EEG-based CAD system to assist the accurate diagnosis of psychiatric disorders (Bzdok and Meyer-Lindenberg, 2018; Fazel and O’Reilly, 2020; Rutherford, 2020; Koutsouleris et al., 2021). In particular, it is important to use neurophysiologically interpretable EEG biomarkers in the development of an EEG-based CAD system to improve its reliability, as in deep learning (Sturm et al., 2016). In this study, we introduced low frequency EEG features that were closely associated with the neurophysiological characteristics of PTSD patients (Franke et al., 2016; Vergara et al., 2017; Newson and Thiagarajan, 2019), and thereby not only the proposed low frequency EEG biomarkers could significantly improve the performance of the CAD system, but also they were neurophysiologically interpretable. To evaluate the potential of low frequency EEG features for the performance improvement of the CAD system, we used a traditional machine learning approach using PSDs as features and SVM as a classifier, and the improved classification accuracy of 86.61% would not be still enough to be used in clinics. Even though we also used 3 different CNN-based deep learning algorithms for the diagnosis of PTSD patients as mentioned in the method section, we could not obtain a comparable classification accuracy to that obtained using the SVM approach (73.13% for Shallow ConvNet, 76.15% for EEGNet, and 77.03% for 13-Layers CNN). This result would be derived due to a relatively small number of samples (data amount) used in deep learning algorithms; traditional machine-learning models show better performance than that of deep learning models with relatively small samples (Kumar and Manash, 2019). Therefore, we will keep trying to improve the diagnosis accuracy by introducing more advanced algorithms, such as data augmentation to compensate a small number of samples in our future studies (Wang et al., 2018), thereby developing a clinically usable CAD system for PTSD patients.

### Limitations

First, since all recruited PTSD patients were on medication, we could not control the compounding effects from medications. It has been reported that EEG characteristics can be changed by medications. For example, antipsychotics increased slow waves, such as delta and theta (Amann et al., 2003), antidepressants modified alpha patterns (Bruder et al., 2008), and sedative-hypnotics enhanced low frequency power (< 15 Hz) (Ferri et al., 2017). All psychotic medications tend to increase PSD values in particular for relatively low frequency bands, but delta and theta PSDs for PTSD patients were still significantly lower than those of HC, as shown in Figure 3. Therefore, it could be reasonably thought that delta and theta PSDs of PTSD patients could be increased by medications, but which did not reach those of HC. Thereby, significant difference between two groups was still kept in terms of low frequency PSD values, and they were used as key features for accurate diagnosis. Our previous study also used same types of medications used in this study, i.e., antipsychotics, antidepressants, and sedative-hypnotics (Kim Y. W. et al., 2020). Because patients recruited in the previous study were independent from those recruited in the present study, it is impossible to directly compare the results of the two studies. However, assuming that similar medication impacts occurred on EEG characteristics due to using same types of medications for both groups of PTSD patients, it can be reasonably thought that the enhanced classification performance in this study would be caused by using optimal PSD features as compared to the previous study, not medication effects. Further studies should follow with drug-naïve PTSD patients to accurately investigate the medication impact on the development of an EEG-based CAD system, which can also allow for PTSD diagnosis at initial screening stages. Second, even though we controlled other psychiatric illness, we did not control comorbid depression. Third, we used a total of 40.96-s EEG data measured using 64 electrodes for data analysis (e.g., source estimation). It was reported that reliable source signal estimation can be possible using the EEG data acquired from more than 60 channels (Michel et al., 2004), and also reliable resting-state EEG data analysis with a data length of more than 40 s (Gudmundsson et al., 2007). However, since it is obvious that use of longer EEG data measured from a high-density EEG system with more channels (e.g., > 128) can allow for more reliable EEG data analysis, including source imaging (Haartsen et al., 2020), using such the high-density EEG data is necessary to improve the reliability and accuracy of EEG data analysis in future studies. Fourth, PTSD patients showed different EEG characteristics according to gender. For example, female patients showed enhanced alpha asymmetry than female HC (Metzger et al., 2004), while male patients showed decreased source activities in theta band and decreased alpha PSD as compared to male HC (Jokić-begić and Begić, 2003; Todder et al., 2012). Therefore, diagnostic performance could be further improved by considering gender-specific EEG features, which would be one of the interesting future topics.

## Conclusion

We investigated optimal PSD frequency bands to improve the classification performances of a resting-state EEG-based CAD system for precise PTSD diagnosis. Low-frequency PSD features in delta and theta frequency bands showed significantly higher classification performances than relatively high-frequency PSD features (alpha, low-beta, high-beta, and gamma), and the best classification performance (86.61% and AUC of 0.93) was obtained when using delta PSD features. In addition, most meaningful features were extracted from fronto-tempora areas, which coincided with the neurophysiological findings of previous EEG-based PTSD studies. Although the present study showed relatively high classification performances between PTSD patients and HC, there is still room to improve the classification performance by introducing novel feature extraction and classification methods based on deep learning algorithms, which could be interesting future research topics.

## Data Availability Statement

The raw data supporting the conclusions of this article will be made available by the authors, without undue reservation.

## Ethics Statement

The studies involving human participants were reviewed and approved by the Institutional Review Board of Inje University Ilsan Paik Hospital (2015-09-018). The patients/participants provided their written informed consent to participate in this study.

## Author Contributions

MS, S-HL, and H-JH: conception and design of the work and interpretation of data. MS and S-HL: data acquisition and analysis. MS, C-HI, S-HL, and H-JH: writing (original draft preparation and editing). All authors read and contributed to the final version of the manuscript.

## Conflict of Interest

The authors declare that the research was conducted in the absence of any commercial or financial relationships that could be construed as a potential conflict of interest.

## Publisher’s Note

All claims expressed in this article are solely those of the authors and do not necessarily represent those of their affiliated organizations, or those of the publisher, the editors and the reviewers. Any product that may be evaluated in this article, or claim that may be made by its manufacturer, is not guaranteed or endorsed by the publisher.
