# Supplementary material for: Enhanced Performance by Interpretable Low-Frequency Electroencephalogram Oscillations in the Machine Learning-Based Diagnosis of Post-traumatic Stress Disorder
Source: Front Neuroinform. 2022 Apr 26;16:811756. doi: 10.3389/fninf.2022.811756 (PMC9094422; doi:10.3389/fninf.2022.811756)
Supplement: Supplementary file 1 [file Presentation_1.PDF]

Supplementary Material

Supplementary Table 1. Abbreviations used in the manuscript

Full name	Abbreviation	Full name	Abbreviation
Post-traumatic stress disorder	PTSD	Healthy controls	HC
Electroencephalography	EEG	Computer-aided diagnosis	CAD
Power spectrum density	PSD	Traumatic brain injury	TBI
magnetoencephalographic	MEG	Area-under-the-curve	AUC
Event-related potential	ERP	Diagnostic and Statistical Manual of Mental Disorders, 4th edition	DSM-IV
Beck Anxiety Inventory	BAI	Impact of Event Scale-Revised	IES-R
Beck Depression Inventory	BDI	Electrocardiogram	ECG
Boundary element method	BEM	Regions of interest	ROI
Weighted minimum-norm estimation	wMNE	Principal component analysis	PCA
Sequential backward selection	SBS	Receiver operating characteristics	ROC

Supplementary Table 2. Regions of interest (ROI) based on the Desikan-Killiany atlas, and their corresponding mean and standard deviation of variances explained by 1st principal component for each group with *p*-values for group differences of variances obtained using independent *t*-test with Bonferroni correction. ROIs with gray color were excluded for feature extraction because they showed statistical difference between the two groups in terms of the variances of 1st principal components.

Num.	ROI	PTSD	HC	*p*-value
1	Bankssts L	0.84 ± 0.11	0.87 ± 0.11	0.278
2	Bankssts R	0.90 ± 0.08	0.91 ± 0.10	0.602
3	Caudal anterior cingulate L	0.86 ± 0.09	0.87 ± 0.10	0.836
4	Caudal anterior cingulate R	0.85 ± 0.10	0.84 ± 0.11	0.795
5	Caudal middle frontal L	0.67 ± 0.15	0.66 ± 0.15	0.772
6	Caudal middle frontal R	0.66 ± 0.13	0.67 ± 0.13	0.875
7	Cuneus L	0.77 ± 0.14	0.73 ± 0.11	0.220
8	Cuneus R	0.73 ± 0.14	0.69 ± 0.10	0.206
9	Entorhinal L	0.97 ± 0.04	0.96 ± 0.06	0.579
10	Entorhinal R	0.97 ± 0.04	0.97 ± 0.05	0.979
11	Frontal pole L	0.93 ± 0.07	0.89 ± 0.10	0.047
12	Frontal pole R	0.90 ± 0.09	0.88 ± 0.10	0.370
13	Fusiform L	0.77 ± 0.11	0.77 ± 0.11	0.875
14	Fusiform R	0.77 ± 0.11	0.84 ± 0.10	0.006
15	Inferior parietal L	0.70 ± 0.16	0.71 ± 0.15	0.878
16	Inferior parietal R	0.65 ± 0.17	0.74 ± 0.17	0.019
17	Inferior temporal L	0.70 ± 0.13	0.73 ± 0.14	0.483
18	Inferior temporal R	0.73 ± 0.12	0.79 ± 0.13	0.047
19	Insula L	0.82 ± 0.11	0.77 ± 0.11	0.020
20	Insula R	0.85 ± 0.09	0.80 ± 0.11	0.026
21	Isthmus cingulate L	0.86 ± 0.10	0.90 ± 0.08	0.020
22	Isthmus cingulate R	0.87 ± 0.10	0.92 ± 0.07	0.008
23	Lateral occipital L	0.71 ± 0.17	0.71 ± 0.14	0.878
24	Lateral occipital R	0.67 ± 0.15	0.66 ± 0.14	0.795
25	Lateral orbitofrontal L	0.85 ± 0.12	0.81 ± 0.12	0.139
26	Lateral orbitofrontal R	0.85 ± 0.10	0.84 ± 0.09	0.688
27	Lingual L	0.80 ± 0.14	0.80 ± 0.12	0.982
28	Lingual R	0.80 ± 0.11	0.79 ± 0.10	0.856
29	Medial orbitofrontal L	0.84 ± 0.10	0.82 ± 0.08	0.444
30	Medial orbitofrontal R	0.86 ± 0.10	0.88 ± 0.06	0.641
31	Middle temporal L	0.65 ± 0.13	0.66 ± 0.14	0.772
32	Middle temporal R	0.66 ± 0.14	0.69 ± 0.15	0.483
33	Paracentral L	0.78 ± 0.12	0.84 ± 0.12	0.026

34	Paracentral R	0.78 ± 0.11	0.84 ± 0.10	0.016
35	Para hippocampal L	0.94 ± 0.06	0.95 ± 0.05	0.872
36	Para hippocampal R	0.94 ± 0.07	0.96 ± 0.03	0.020
37	Pars opercularis L	0.77 ± 0.13	0.76 ± 0.12	0.856
38	Pars opercularis R	0.80 ± 0.12	0.74 ± 0.13	0.038
39	Pars orbitalis L	0.92 ± 0.08	0.92 ± 0.09	0.856
40	Pars orbitalis R	0.90 ± 0.08	0.83 ± 0.13	0.006
41	Pars triangularis L	0.91 ± 0.09	0.91 ± 0.08	0.982
42	Pars triangularis R	0.87 ± 0.11	0.85 ± 0.08	0.406
43	Pericalcarine L	0.85 ± 0.12	0.84 ± 0.10	0.643
44	Pericalcarine R	0.85 ± 0.12	0.82 ± 0.09	0.220
45	Post central L	0.54 ± 0.15	0.57 ± 0.16	0.483
46	Postcentral R	0.54 ± 0.14	0.55 ± 0.15	0.856
47	Posterior cingulate L	0.85 ± 0.11	0.92 ± 0.04	0.000
48	Posterior cingulate R	0.85 ± 0.12	0.92 ± 0.04	0.000
49	Precentral L	0.54 ± 0.15	0.56 ± 0.16	0.751
50	Precentral R	0.54 ± 0.14	0.52 ± 0.14	0.483
51	Precuneus L	0.69 ± 0.12	0.76 ± 0.12	0.018
52	Precuneus R	0.69 ± 0.11	0.78 ± 0.11	0.000
53	Rostral anterior cingulate L	0.89 ± 0.10	0.86 ± 0.09	0.406
54	Rostral anterior cingulate R	0.88 ± 0.11	0.88 ± 0.08	0.915
55	Rostral middle frontal L	0.63 ± 0.16	0.58 ± 0.14	0.201
56	Rostral middle frontal R	0.63 ± 0.16	0.58 ± 0.13	0.139
57	Superior frontal L	0.54 ± 0.17	0.50 ± 0.12	0.278
58	Superior frontal R	0.55 ± 0.15	0.51 ± 0.12	0.389
59	Superior parietal L	0.63 ± 0.14	0.65 ± 0.13	0.715
60	Superior parietal R	0.63 ± 0.13	0.67 ± 0.15	0.220
61	Superior temporal L	0.66 ± 0.14	0.66 ± 0.14	0.940
62	Superior temporal R	0.70 ± 0.12	0.67 ± 0.12	0.483
63	Supramarginal L	0.68 ± 0.14	0.70 ± 0.16	0.751
64	Supramarginal R	0.68 ± 0.14	0.73 ± 0.14	0.150
65	Temporal pole L	0.97 ± 0.04	0.94 ± 0.09	0.026
66	Temporal pole R	0.96 ± 0.06	0.94 ± 0.07	0.304
67	Transverse temporal L	0.88 ± 0.09	0.88 ± 0.10	0.909
68	Transverse temporal R	0.89 ± 0.09	0.88 ± 0.10	0.856

Supplementary Table 3. Classification accuracies of all 30 feature sets.

Feature set	Balanced accuracy	Sensitivity	Specificity	Num. of Features
Delta	86.61	87.01	86.21	18
Theta	82.06	77.92	86.21	35
Alpha	72.79	76.62	68.97	46
Low Beta	69.36	81.82	56.90	38
High Beta	54.50	88.31	20.69	1
Gamma	50.00	0.00	100.00	7
D+T	86.39	83.12	89.66	95
D+A	86.17	84.42	87.93	71
D+LB	86.17	84.42	87.93	96
D+HB	86.17	84.42	87.93	77
D+G	85.96	85.71	86.21	3
T+A	82.06	77.92	86.21	62
T+LB	83.36	80.52	86.21	57
T+HB	83.36	80.52	86.21	62
T+G	83.36	80.52	86.21	58
A+LB	72.79	76.62	68.97	63
A+HB	73.44	77.92	68.97	84
A+G	74.09	79.22	68.97	97
LB+HB	70.22	81.82	58.62	81
LB+G	70.22	81.82	58.62	26
HB+G	55.36	88.31	22.41	28
D+T+A	85.74	81.82	89.66	135
T+A+LB	82.71	79.22	86.21	106
A+LB+HB	74.96	79.22	70.69	99
LB+HB+G	71.31	85.71	56.90	192
D+T+A+LB	85.96	85.71	86.21	144
T+A+LB+HB	82.71	79.22	86.21	147
A+LB+HB+G	73.88	80.52	67.24	167
D+T+A+LB+HB	84.66	83.12	86.21	242
D+T+A+LB+HB+G	86.39	83.12	89.66	215

Num. of Features – number of features
